# Community-based interventions for the prevention and control of helmintic neglected tropical diseases

**DOI:** 10.1186/2049-9957-3-23

**Published:** 2014-07-31

**Authors:** Rehana A Salam, Hasina Maredia, Jai K Das, Zohra S Lassi, Zulfiqar A Bhutta

**Affiliations:** 1 Division of Women and Child Health, The Aga Khan University, Karachi 74800, Pakistan; 2 Brown University, Providence, RI, USA; 3 Center of Excellence in Women & Child Health, The Aga Khan University, Karachi, Pakistan; 4 Center for Global Child Health Hospital for Sick Children, Toronto, Canada

**Keywords:** Neglected tropical diseases, Soil-transmitted helminthiasis, Community-based interventions

## Abstract

In this paper, we aim to systematically analyze the effectiveness of community-based interventions (CBIs) for the prevention and control of helminthiasis including soil-transmitted helminthiasis (STH) (ascariasis, hookworms, and trichuriasis), lymphatic filariasis, onchocerciasis, dracunculiasis, and schistosomiasis. We systematically reviewed literature published before May 2013 and included 32 studies in this review. Findings from the meta-analysis suggest that CBIs are effective in reducing the prevalence of STH (RR: 0.45, 95% CI: 0.38, 0.54), schistosomiasis (RR: 0.40, 95% CI: 0.33, 0.50), and STH intensity (SMD: −3.16, 95 CI: −4.28, −2.04). They are also effective in improving mean hemoglobin (SMD: 0.34, 95% CI: 0.20, 0.47) and reducing anemia prevalence (RR: 0.90, 95% CI: 0.85, 0.96). However, it did not have any impact on ferritin, height, weight, low birth weight (LBW), or stillbirths. School-based delivery significantly reduced STH (RR: 0.49, 95% CI: 0.39, 0.63) and schistosomiasis prevalence (RR: 0.50, 95% CI: 0.33, 0.75), STH intensity (SMD: −0.22, 95% CI: −0.26, −0.17), and anemia prevalence (RR: 0.87, 95% CI: 0.81, 0.94). It also improved mean hemoglobin (SMD: 0.24, 95% CI: 0.16, 0.32). We did not find any conclusive evidence from the quantitative synthesis on the relative effectiveness of integrated and non-integrated delivery strategies due to the limited data available for each subgroup. However, the qualitative synthesis from the included studies supports community-based delivery strategies and suggests that integrated prevention and control measures are more effective in achieving greater coverage compared to the routine vertical delivery, albeit it requires an existing strong healthcare infrastructure. Current evidence suggests that effective community-based strategies exist and deliver a range of preventive, promotive, and therapeutic interventions to combat helminthic neglected tropical diseases (NTDs). However, there is a need to implement and evaluate efficient integrated programs with the existing disease control programs on a larger scale throughout resource-limited regions especially to reach the unreachable.

## Multilingual abstracts

Please see Additional file 1 for translations of the abstract into the six official working languages of the United Nations.

## Introduction

Helminths (Greek, meaning ‘worms’) are parasitic worms that have been harboring in humans throughout the ages. These are classified as **nematodes or roundworms** and include soil-transmitted helminths (ascariasis, hookworms, and trichuriasis) and filarial (causing lymphatic filariasis [LF] and onchocerciasis), whereas the **platyhelminthes or flatworms** include the flukes (schistosomes) and tapeworms. As discussed in Paper 1 [1], helminthic infections are a prominent subgroup within neglected tropical diseases (NTDs), primarily perpetuated due to a lack of access to safe water and sanitation. The most common helminths are the soil-transmitted helminthiasis (STH), followed by schistosomiasis and LF. These are often co-infectious, although the biology of each disease differs. These infections disproportionately affect children, pregnant women, and young adults resulting in serious chronic health conditions including malnutrition, physical and intellectual growth retardations in children, and adverse maternal, perinatal and delivery outcomes among pregnant women [2-4]. Onchocerciasis is a leading cause of blindness and skin disease, while LF is a major cause of limb and genital deformities. For a more thorough discussion on the epidemiology and burden of each of these diseases, please refer to our previous publication [1].

Mass drug administration (MDA) with anthelminthics has been a major approach to combat human helminthiasis while parallel interventions such as micronutrient supplementation, especially iron, to combat associated anemia has also been widely evaluated for effectiveness [5]. Sanitation and education are also recommended, however, these are not always feasible in resource-limited settings. All these strategies can be effectively administered via community delivery platforms. Child health days, micronutrient supplementation, vaccination programs, and school-based programs provide a potential entry point for periodic deworming and health education in a more cost-effective manner [6]. In this paper, we aim to systematically analyze the effectiveness of community-based interventions (CBIs) for the prevention and control of helminthiasis including STH (ascariasis, hookworms, and trichuriasis), LF, onchocerciasis, dracunculiasis, and schistosomiasis.

## Methods

We systematically reviewed literature published before May 2013 to identify studies on the effectiveness of the community-based delivery of interventions highlighted in our conceptual framework [7] for the outlined helminthic diseases. Our priority was to select existing randomized, quasi-randomized, and before-and-after studies in which the intervention was delivered within community settings and the reported outcomes were relevant to the diseases under review. A separate search strategy was developed for each disease using appropriate keywords, medical subject headings (MeSH), and free text terms. Searches were conducted in the PubMed, Cochrane Libraries, Embase, and the World Health Organization (WHO) Regional Databases. Studies that met the inclusion criteria were selected and double data was abstracted on a standardized abstraction sheet. Quality assessment of the included randomized controlled trials (RCTs) was done using the Cochrane risk of bias assessment tool [8]. The outcomes of interest assessed for each of the above diseases are outlined in Table 1. We conducted a meta-analysis for individual studies using the software Review Manager 5.1. Pooled statistics were reported as the relative risk (RR) for categorical variables and standard mean difference (SMD) for continuous variables between the experimental and control groups with 95% confidence intervals (CIs). We also attempted to qualitatively synthesize the findings reported in the included studies for other pragmatic parameters identified in our conceptual framework including intervention coverage, challenges/barriers, enabling factors, aspects related to integrated delivery, monitoring, and evaluations and equity. The detailed methodology is described in Paper 2 of this series [7].

**Table 1:** Outcomes analyzed

***Outcomes***	**Outcomes analyzed**
**Morbidity**	• Hookworm prevalence and intensity
	• Ascaris prevalence and intensity
	• Trichuris prevalence and intensity
	• *Schistosoma haematobium* prevalence
	• *Schistosoma mansoni* prevalence
	• *Schistosoma japonicum* prevalence
**Anthropometry**	• Weight
	• Height
**Hematologic**	• Prevalence of anemia
	• Mean hemoglobin
	• Serum ferritin
**Birth outcomes**	• Birth weight
	• Low birth weigh
	• Very low birth weight
	• Stillbirths

## Review

**Figure 1:**
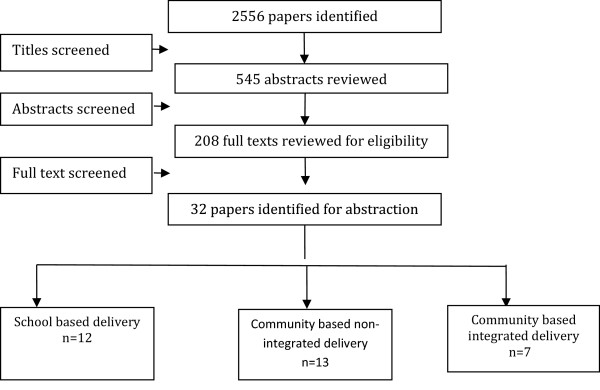
Search flow diagram.

We identified 2,556 titles from the searches conducted in all databases. After screening titles and abstracts, 208 full texts were reviewed, of which 32 studies (17 RCTs, two quasi, and 13 before-and-after studies) were included in the review (see Figure 1). The characteristics of the included studies are summarized in Table 2. Of these 32 studies, four could not be included in the meta-analysis; one study [9] did not report any outcome of interest, poolable data was not available in two studies [10,11], while one [12] did not have a suitable control group for comparison. We did not find any quantifiable data from the studies on dracunculiasis, LF, and onchocerciasis to be included in the pooled analysis. For the 17 RCTs included in this review, randomization was adequate in 15 studies, allocation was concealed in six, and adequate sequence generation was also done in six. All studies provided insufficient information on selective reporting which limited us from making any judgment (see Table 3).

**Table 2:** Characteristics of the included studies

***Study***	***Study design***	***Country***	***Intervention***	***Target population***	***Integrated/Non-Integrated***
Adam 1995 [13]	RCT	Kenya	Three doses of 400 mg ALB vs. identical placebo on three consecutive school days	Children aged 5–10 years infected with hookworms or ascariasis	School-based
Ageel 1997 [33]	Pre/post	Saudi Arabia	One oral dose of PZQ (40 mg/kg) + snail/vector control + health education + community participation	General population infected with schistosomiasis	PHC
Albonico 2002 [14]	RCT	Tanzania	MBZ 500 mg vs. pyrantel-oxantel (10 mg/kg) vs. placebo	Children aged 6–9 years	School-based
Bausch 1995 [35]	Quasi	Cameroon	Health education + PZQ	Children aged 5–15 years with schistosomiasis	PHC + school-based
Beasely 1999 [25]	RCT	Tanzania	Single dose of 400 mg ALB + PPZQ (40 mg/kg) vs. placebo	Infected children aged 7–12 years	Non-integrated
Bhargawa 2003 [15]	RCT	Tanzania	ALB 400 mg + PZQ single 40 mg/kg vs. placebo	Infected children aged 9–15 years	School-based
Christian 2009 [36]	RCT	Nepal	ALB 40 mg in 2^nd^ + 3^rd^ trimester	Pregnant women	Integrated with routine ANC
Dossa 2001 [26]	RCT	Benin	Iron (Fe) (60 mg/day) + ALB (200 mg for 3 days) vs. FE + placebo vs. ALB + placebo vs. placebo 10 months F/V	Children aged 3–5 years	Non-integrated
Engels 1993 [11]	Pre/post	Burundi	Single dose PZQ (40 mg/kg), 50–150 mg levamisole or 500 mg MBL or PZQ (20 mg/kg) + snail vector control + education	General population	PHC
Gryseels 1991 [23]	Pre/post	Burundi	PZQ (40 mg/kg)	General population	Non-integrated
Gundersen 1990 [34]	Pre/post	Ethiopia	Vector snail control + 20 mg oxamniquine followed by 40 mg the next day	General population	PHC
Guyatt 2001 [16]	Pre/post	Tanzania	Single dose 400 mg ALB + PZQ 40 mg/kg	School children aged 8–14 years	School-based
Halwindi 2011 [27]	RCT	Zambia	Facility vs. community detected and treatment + training + education + MBZ	Children aged 12–months (preventive)	Non-integrated
Haque 2010 [28]	RCT	Bangladesh	400 mg ALB + B-carotene (2 doses) vs. ALB + placebo vs. B-carotene + placebo vs. placebo + placebo	Infected children aged 24–60 months	Non-integrated
Hathirat 1992 [24]	RCT	Thailand	Fe 50 mg FeSo 4 daily for 2 weeks then 100 mg for 14wks vs. anthelminthic	Children aged 9–11 years	School-based
Kosinski 2012 [31]	Pre/post	Ghana	Water recreation area + PZQ	Children	Non-integrated
Koukounari 2006 [17]	Pre/post	Uganda	PZQ	Children aged 7–14 years	School-based
Koukounari 2007 [18]	Pre/post	Burkina Faso	PZQ 40 mg/kg + ALB 400 mg	Children aged 6–14 years	School-based
Larocque 2006 [37]	RCT	Peru	MBZ 500 mg single dose + 60 mg FE daily vs. placebo + FE	Pregant women aged 18–44 years in the 2^nd^ trimester	Integrated with routine ANC
Ndibazza 2010 [38]	RCT	Uganda	ALB (400 mg) vs. PZQ 40 mg/kg vs. ALB + PZQ vs. placebo	Pregnant women	Integrated with routine ANC
Nsowah-Nuamah 2001 [12]	Pre/post	Ghana	PZQ 40 mg/kg + passive edu vs. PZQ + no education vs. PZQ + active edu vs. community mobilization	Those aged 5 years and above	Non-integrated
Olds 1999 [29]	RCT	China and Philippines	ALB + PZQ vs. PZQ + ALB placebo vs. ALB + PZQ placebo vs. placebo + placebo	Infected school aged children (4–18 years)	Non-integrated
Palupi 1997 [30]	RCT	Indonesia	FE + ALB 400 mg once a week vs. FE once a week + placebo vs. placebo alone	Children aged 2–5 years	Non-integrated
Phuc 2009 [40]	Pre/post	Vietnam	IEC materials + staff training + Ferrous Sulphate/ folic acid (200 mg) + ALB (400 mg)	Women of reproductive age (16–45 years)	Non-integrated
Rohner 2010 [19]	RCT	Cote d’Ivoire	Iron fortified biscuits (20 mg fe/day) + IPT-SP (500 mg sulphadoxine + 25 mg pyrimethamine) + anthelminthic (400 mg-single) + PZQ (single – 40 mg/kg) in different combinations	School children aged 6–14 years	School-based
Savioli 1989 [9]	Pre/post	Tanzania	PZQ	Children	School-based
Sungthong 2002 [20]	RCT	Thailand	Deworming (400 mg ALB single) + daily or weekly iron	Children aged 6–13 years	School-based
Sinoun 2007 [10]	Pre/post	Cambodia	Universal chemotherapy with PZQ (40 mg/kg)	General population	Non-integrated
Taylor 2001 [21]	RCT	South Africa	ALB (400 mg) + PZQ (40 mg/kg) + FE fumigate 200 mg weekly vs. ALB (400 mg) + PZQ (40 mg/kg) + placebo weekly vs. ALB (400 mg) daily + PZQ (40 mg/kg) + FE 200 mg weekly vs. ALB (400 mg) + PZQ (40 mg/kg) + placebo weekly vs. placebos + FE 200 mg weekly vs. placebos	Children aged 6–15 years	School-based
Torlesse 2001 [39]	RCT	Sierra Leone	FE + ALB vs. FE alone vs. ALB alone vs. placebo	Pregnant women (10–14 weeks)	Integrated with routine ANC
Wang 2009 [32]	Quasi	China	Health education + vector control + removing cattle + latrines + PZQ	General population	Non-integrated
Zhang 2007 [22]	Pre/post	Uganda	PZQ (40 mg/kg) + ALB(400 mg) + education	School children and adults	Non-integrated

**Table 3:** Quality assessment of the included RCTs

***Study***	***Randomization***	***Sequence generation***	***Allocation concealment***	***Blinding of participants***	***Blinding of assessors***	***Selective reporting***
Larocque 2006 [37]	Done	Done	Done	Done	Done	No
Ndibazza 2010 [38]	Done	Done	Done	Done	Done	No
Torlesse 2001 [39]	Done	Done	Not clear	Not clear	Not clear	Yes
Haque 2010 [28]	Done	Not clear	Not clear	Done	Not clear	No
Albonico 2002 [14]	Done	Done	Done	Done	Done	No
Taylor 2001 [21]	Done	Not clear	Not clear	Done	Done	Not clear
Adams 1994 [13]	Done	Not clear	Not clear	Done	Not clear	No
Beasley 1999 [25]	Done	Not computerized but done	Not clear	Not done	Done	No
Christian 2009 [36]	Not clear	Not clear	Not clear	Not Clear	Not Clear	Not clear
Halwindi 2011 [27]	Not clear	Not clear	Not applicable	Not applicable	Not clear	No
Olds 1999 [29]	Done	Not clear	Done	Done	Done	Not clear
Rohner 2010 [19]	Done	Not clear	Done	Done	Done	No
Bhargava 2003 [15]	Done	Not clear	Not clear	Not clear	Not clear	No
Palupi 1997 [30]	Done	Not clear	Not clear	Not clear	Not clear	No
Dossa 2001 [26]	Done	Not clear	Not clear	Done	Done	Yes
Hathirat 1992 [24]	Done	Not clear	Not clear	Done	Done	No
Sungthong 2002 [20]	Done	Done	Done	Done	Done	No

Included studies mainly focused on community-based MDA, which involved preventive chemotherapy in 19 of the studies and treatment after confirmed diagnosis in 11 studies. A school-based delivery strategy was the most common delivery strategy used in 12 [9,13-21,24,25] studies targeting children aged five to 15 years, thirteen [10,12,22,23,25-32,40] studies were non-integrated vertically delivered interventions, seven [11,33,34,36-39] were integrated with primary healthcare (PHC), and routine antenatal care (ANC) services. Almost all the studies had a component on health education to promote general hygiene and sanitation along with the drug administration. Other co-interventions included iron and β-carotene supplementation, snail control, constructing latrines, eliminating cattle from the residential areas, staff training, and community mobilization. One study [31] assessed the effectiveness of constructing a water recreation area in the community using a local lake to prevent transmission of schistosomiasis among school children. Most of the studies provided combined drug treatment for the prevention and treatment of hookworms, ascariasis, trichuriasis, and schistosomiasis with the treatment regimen including administration of albendazole (ALB) 400 mg for STH and praziquantel (PZQ) 40 mg/kg for schistosomiasis, while mebendazole (MBZ) 500 mg was used in four studies [11,14,27,37] and one study [14] also administered pyrantel-oxantel (10 mg/kg). The frequency of treatment varied according to the prevalence in the intervention area. We also attempted to conduct a subgroup analysis for the relative effectiveness of preventive and therapeutic drug administration, integrated and non-integrated delivery strategies, and evidence from RCTs/quasi and pre-post studies separately, where possible, and reported the results accordingly. The results are summarized in Tables 4 and 5.

**Table 4:** Results for the overall and subgroup analysis according to type of study and treatment

**Outcomes**	**Estimates (95% CI)**				
	**Combined**	**RCTs**	**Pre-post studies**	**Preventive**	**Therapeutic**
**STH Prevalence (RR)**	**0.47 [0.41, 0.54]**	**0.47 [0.41, 0.55]**	**0.51 [0.40, 0.65]**	**0.43 [0.31, 0.59]**	**0.52 [0.44, 0.61]**
	45 datasets from 12 studies	40 datasets from 9 studies	5 datasets from 3 studies	22 datasets from 7 studies	23 datasets from 5 studies
Hookworm	**0.40 [0.31, 0.52]**	**0.41 [0.32, 0.54]**	**0.39 [0.23, 0.65]**	**0.26 [0.14, 0.45]**	**0.60 [0.46, 0.78]**
	15 datasets, 10 studies	13 datasets from 8 studies	2 datasets from 2 studies	8 datasets from 6 studies	7 datasets from 4 studies
Ascariasis	**0.32 [0.20, 0.51]**	**0.30 [0.18, 0.49]**	**0.61 [0.41, 0.90]**	0.62 [0.38, 1.01]	**0.18 [0.08, 0.40]**
	15 datasets, 9 studies	14 datasets from 8 studies	1 dataset from 1 study	7 datasets from 4 studies	8 datasets from 5 studies
Trichuriasis	**0.66 [0.55, 0.80]**	**0.65 [0.53, 0.79]**	0.97 [0.68, 1.37]	0.57 [0.22, 1.46]	**0.76 [0.66, 0.88]**
	14 datasets, 8 studies	13 datasets from 7 studies	1 dataset from 1 study	6 datasets from 3 studies	8 datasets from 5 studies
**Schistosomiasis Prevalence (RR)**	**0.41 [0.34, 0.50]**	**0.42 [0.33, 0.54]**	**0.37 [0.25, 0.55]**	**0.39 [0.27, 0.55]**	**0.41 [0.30, 0.55]**
	25 datasets, 13 studies	16 datasets from 6 studies	9 datasets from 7 studies	11 datasets from 7studies	14 datasets from 6 studies
***Schistosoma Haematobium***	**0.41 [0.30, 0.57]**	**0.43 [0.29, 0.64]**	**0.33 [0.17, 0.65]**	0.59 [0.35, 1.02]	**0.31 [0.18, 0.52]**
	10 datasets, 8 studies	6 datasets from 4 studies	4 datasets from 4 studies	3 datasets from 3 studies	7 datasets from 5 studies
*SchistosomaJaponicum*	**0.24 [0.07, 0.80]**	**0.24 [0.07, 0.80]**	No studies	**0.11 [0.05, 0.26]**	0.30 [0.07, 1.31]
	4 datasets, 2 studies	4 datasets from 2 studies		1 dataset from 1 study	3 datasets from 1 study
*SchistosomaMansoni*	**0.48 [0.36, 0.64]**	**0.55 [0.38, 0.81]**	**0.39 [0.25, 0.62]**	**0.37 [0.24, 0.58]**	0.67 [0.45, 1.01]
	11 datasets, 7 studies	6 datasets from 2 studies	5 datasets from 5 studies	7 datasets from 5 studies	4 datasets from 2 studies
**STH intensity (SMD)**	**−3.16 [−4.28, −2.04]**	**−5.29 [−9.22, −1.36]**	**−0.22 [−0.26, −0.17]**	**−0.22 [−0.26, −0.17]**	**−5.29 [−9.22, −1.36]**
	5 datasets, 3 studies	3 datasets from 1 study	2 datasets from 2 studies	2 datasets from 2 studies	3 datasets from 1 study
Hookworm	**−2.22 [−3.27, −1.17]**	**−6.79 [−7.44, −6.14]**	**−0.22 [−0.26, −0.17]**	**−0.22 [−0.26, −0.17]**	**−6.79 [−7.44, −6.14]**
	3 datasets, 03 studies	1 dataset from 1 study	2 datasets from 2 studies	2 datasets from 2 studies	1 dataset from 1 study
Ascariasis	**−7.25 [−7.93, −6.56]**	**−7.25 [−7.93, −6.56]**	No studies	No studies	**−7.25 [−7.93, −6.56]**
	1 dataset, 1 study	1 dataset from 1 study			1 dataset from 1 study
Trichuriasis	**−1.87 [−2.16, −1.57]**	**−1.87 [−2.16, −1.57]**	No Studies	No studies	**−1.87 [−2.16, −1.57]**
	1 dataset, 01 study	1 dataset from 1 study			1 dataset from 1 study
**Mean hemoglobin (SMD)**	**0.34 [0.20, 0.47]**	**0.43 [0.15, 0.71]**	**0.22 [0.13, 0.31**]	**0.19 [0.12, 0.26]**	1.28 [−0.53, 3.08]
	14 datasets, 12 studies	9 datasets from 7 studies	5 datasets from 5 studies	12 datasets from 10 studies	2 datasets from 2 studies
**Anemia**	**0.90 [0.85, 0.96]**	0.92 [0.85, 1.00]	**0.87 [0.78, 0.97]**	**0.90 [0.85, 0.96]**	0.85 [0.69, 1.06]
	9 datasets, 7 studies	5 datasets from 3 studies	4 datasets from 4 studies	8 datasets from 6 studies	1 dataset from 1 study
**Serum ferritin (SMD)**	2.30 [−1.13, 5.73]	0.06 [−0.07, 0.18]	0.10 [−0.16, 0.37]	0.09 [−0.12, 0.29]	0.07 [−0.07, 0.20]
	4 datasets, 4 studies	2 dataset from 2 studies	2 datasets from 2 studies	3 datasets from 3 studies	1 dataset from 1 study
**Height**	0.01 [−0.10, 0.12]	−0.04 [−0.16, 0.08]	0.23 [−0.02, 0.49]	0.08 [−0.12, 0.28]	−0.02 [−0.15, 0.11]
	4 datasets, 4 studies	3 datasets from 3 studies	1 dataset from 1 study	2 datasets from 2 studies	2 datasets from 2 studies
**Weight**	−0.13 [−0.42, 0.16]	−0.28 [−0.66, 0.11]	0.22 [−0.03, 0.47]	−0.01 [−0.48, 0.46]	−0.35 [−1.13, 0.42]
	4 datasets, 4 studies	3 datasets from 3 studies	1 dataset from 1 study	2 datasets from 2 studies	2 data sets from 2 studies
**Birth outcomes**					
Birth weight	**−9.52 [−13.86, −5.19]**	**−9.52 [−13.86, −5.19]**	No studies	**−9.52 [−13.86, −5.19]**	No study
	6 datasets, 3 studies	6 datasets from 3 studies		6 datasets from 3 studies	
LBW	0.96 [0.78, 1.18]	0.96 [0.78, 1.18]	No studies	0.96 [0.78, 1.18]	No studies
	4 datasets, 2 studies	4 datasets from 2 studies		4 datasets from 2 studies	
VLBW	0.48 [0.19, 1.19]	0.48 [0.19, 1.19]	No studies	0.48 [0.19, 1.19]	No studies
	4 datasets, 2 studies	4 datasets, 2 studies		4 datasets, 2 studies	
Stillbirths	1.54 [0.93, 2.58]	1.54 [0.93, 2.58]	No studies	1.54 [0.93, 2.58]	No studies
	3 datasets, 1 study	3 datasets from 1 study		3 datasets from 1 study	

*estimates in bold are statistically significant.

**Table 5:** Summary estimates for the overall and subgroup analysis for school-based, non-integrated, and integrated delivery strategies

**Outcomes**	**Estimates (95% CI)**			
	**Combined**	**School-based delivery**	**Community-based non-integrated delivery**	**Community-based integrated delivery**
**STH Prevalence (RR)**	**0.45 [0.38, 0.54]**	**0.49 [0.39, 0.63]**	**0.52 [0.41, 0.67]**	**0.30 [0.12, 0.78]**
	45 datasets, 12 studies	19 datasets from 6 studies	16 datasets from 4 studies	9 datasets from 1 study
Hookworm	**0.38 [0.27, 0.53]**	**0.37 [0.22, 0.62]**	**0.49 [0.29, 0.85]**	0.25 [0.04, 1.62]
	15 datasets, 10 studies	7 datasets from 5 studies	5 datasets from 3 studies	3 datasets from 1 study
Ascariasis	**0.32 [0.19, 0.52]**	**0.28 [0.09, 0.89]**	**0.36 [0.18, 0.74]**	**0.28 [0.09, 0.87]**
	15 datasets, 9 studies	4 datasets from 6 studies	6 datasets from 4 studies	3 datasets from 1 studies
Trichuriasis	**0.66 [0.53, 0.82]**	**0.78 [0.67, 0.90]**	**0.76 [0.62, 0.94]**	0.41 [0.07, 2.56]
	14 datasets, 8 studies	4 datasets from 6 studies	5 datasets from 3 study	3 datasets from 1 studies
**Schistosomiasis Prevalence (RR)**	**0.40 [0.33, 0.50]**	**0.50 [0.33, 0.75]**	**0.42 [0.31, 0.57]**	**0.24 [0.11, 0.56]**
	25 datasets, 13 studies	7 datasets from 5 studies	12 datasets from 4 studies	6 datasets from 4 studies
***S. H*** *a* ***ematobium***	**0.40 [0.29, 0.57]**	0.59 [0.35, 1.02]	**0.42 [0.26, 0.70]**	**0.05 [0.01, 0.45]**
	10 datasets, 8 studies	3 datasets from 3 studies	5 datasets from 3 studies	2 datasets from 2 studies
***S. Japonicum***	**0.24 [0.06, 0.87]**	No studies	**0.24 [0.06, 0.87]**	No studies
	4 datasets, 2 studies		4 datasets from 2 studies	
***S. Mansoni***	**0.48 [0.35, 0.65]**	**0.44 [0.27, 0.74]**	0.68 [0.42, 1.10]	**0.38 [0.16, 0.91]**
	11 datasets, 7 studies	4 datasets from 4 studies	3 datasets from 1 study	4 datasets from 2 studies
**STH intensity (SMD)**	**−3.16 [−4.28, −2.04]**	**−0.22 [−0.26, −0.17]**	**−5.29 [−9.22, −1.36]**	No studies
	5 datasets, 3 studies	2 datasets, 2 studies	3 datasets from 1 study	
Hookworm	**−2.22 [−3.27, −1.17]**	**−0.22 [−0.26, −0.17]**	**−6.79 [−7.44, −6.14]**	No studies
	3 datasets, 3 studies	2 datasets, 2 studies	1 dataset from 1 study	
Ascariasis	**−7.25 [−7.93, −6.56]**	No studies	**−7.25 [−7.93, −6.56]**	No studies
	1 dataset, 1 study		1 dataset from 1 study	
Trichuriasis	**−1.87 [−2.16, −1.57]**	No studies	**−1.87 [−2.16, −1.57]**	No studies
	1 dataset, 1 study		1 dataset from 1 study	
**Mean hemoglobin (SMD)**	**0.34 [0.20, 0.47]**	**0.24 [0.16, 0.32]**	0.93 [−0.33, 2.18]	0.09 [−0.01, 0.20]
	14 datasets, 12 studies	7 datasets from 7 studies	3 datasets from 3 studies	4 datasets from 2 studies
**Anemia**	**0.90 [0.85, 0.96]**	**0.87 [0.81, 0.94]**	0.85 [0.69, 1.06]	0.99 [0.90, 1.09]
	9 datasets, 7 studies	5 datasets from 5 studies	1 datasets from 1 study	3 datasets from 1 study
**Serum ferritin (SMD)**	2.30 [−1.13, 5.73]	0.10 [−0.04, 0.25]	No studies	−0.01 [−0.36, 0.34]
	4 datasets, 4 studies	3 datasets from 3 studies		1 dataset from 1 study
**Height**	0.01 [−0.10, 0.12]	0.04 [−0.08, 0.15]	−0.19 [−0.53, 0.14]	No studies
	4 datasets, 4 studies	3 datasets from 3 studies	1 dataset from 1 study	
**Weight**	−0.13 [−0.42, 0.16]	−0.09 [−0.46, 0.27]	−0.27 [−0.60, 0.07]	No studies
	4 datasets, 4 studies	3 datasets from 3 studies	1 dataset from 1 study	
**Birth outcomes**				
Birth weight	**−9.52 [−13.86, −5.19]**	No studies	No studies	**−9.52 [−13.86, −5.19]**
	6 datasets, 3 studies			6 datasets from 3 studies
LBW	0.96 [0.78, 1.18]	No studies	No studies	0.96 [0.78, 1.18]
	4 datasets, 2 studies			4 datasets from 2 studies
VLBW	**0.38 [0.16, 0.87]**	No studies	No studies	**0.38 [0.16, 0.87]**
	4 datasets, 2 studies			4 datasets from 2 studies
Stillbirths	1.54 [0.93, 2.58]	No studies	No studies	1.54 [0.93, 2.58]
	3 datasets, 1 study			3 datasets from 1 study

*Estimates in bold are statistically significant.

### Quantitative synthesis

**Figure 2:**
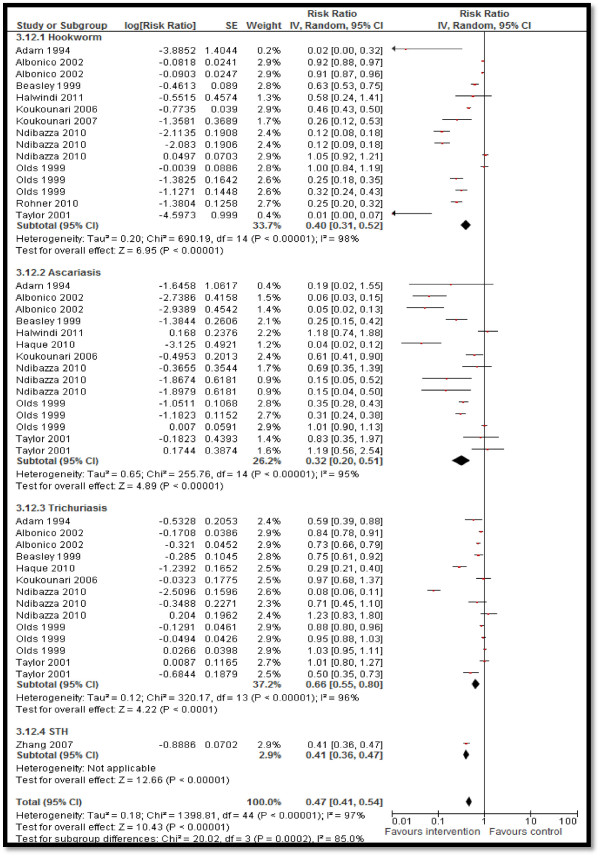
Forest plot for the impact of CBIs on STH prevalence.

**Figure 3:**
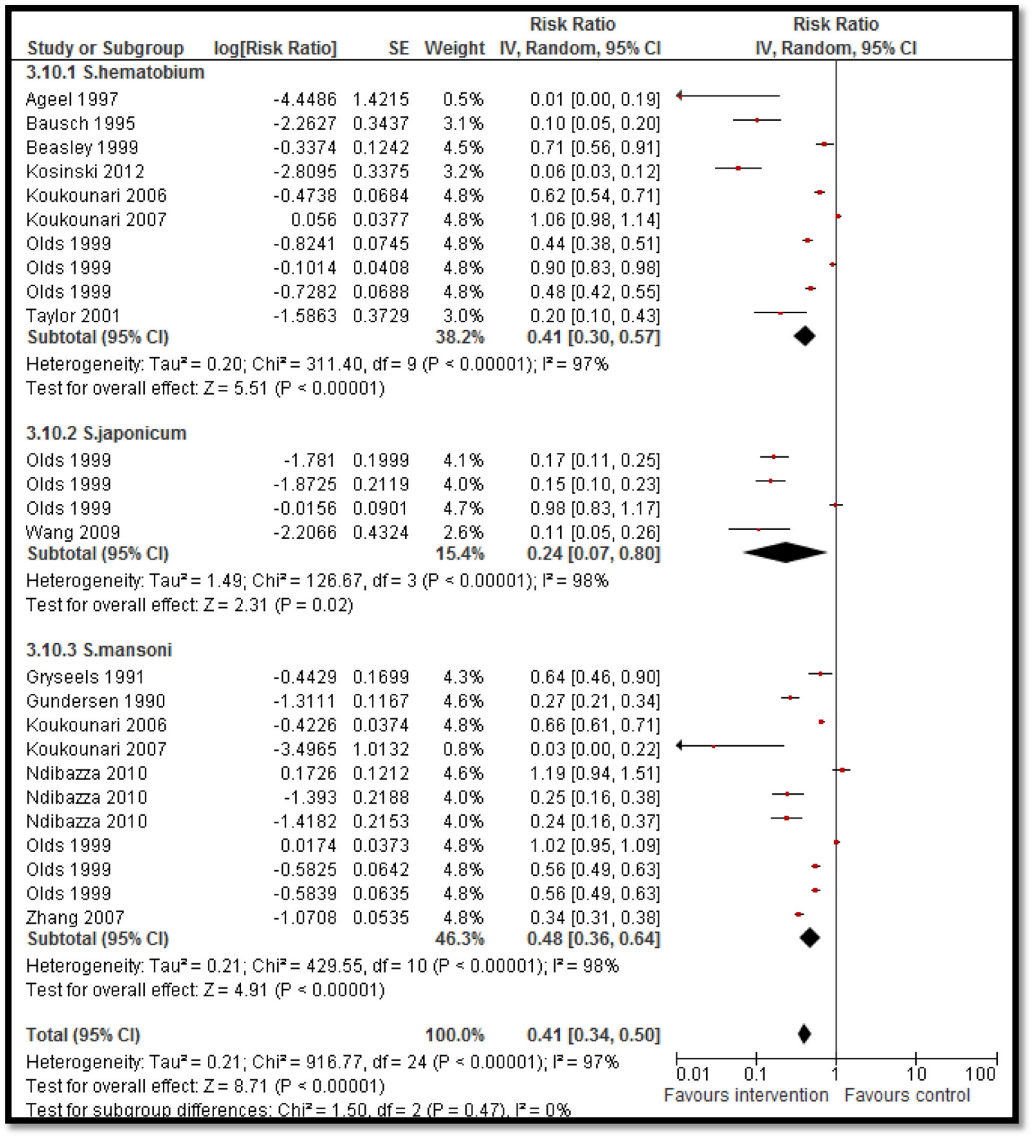
Forest plot for the impact of CBIs on schistosomiasis.

**Figure 4:**
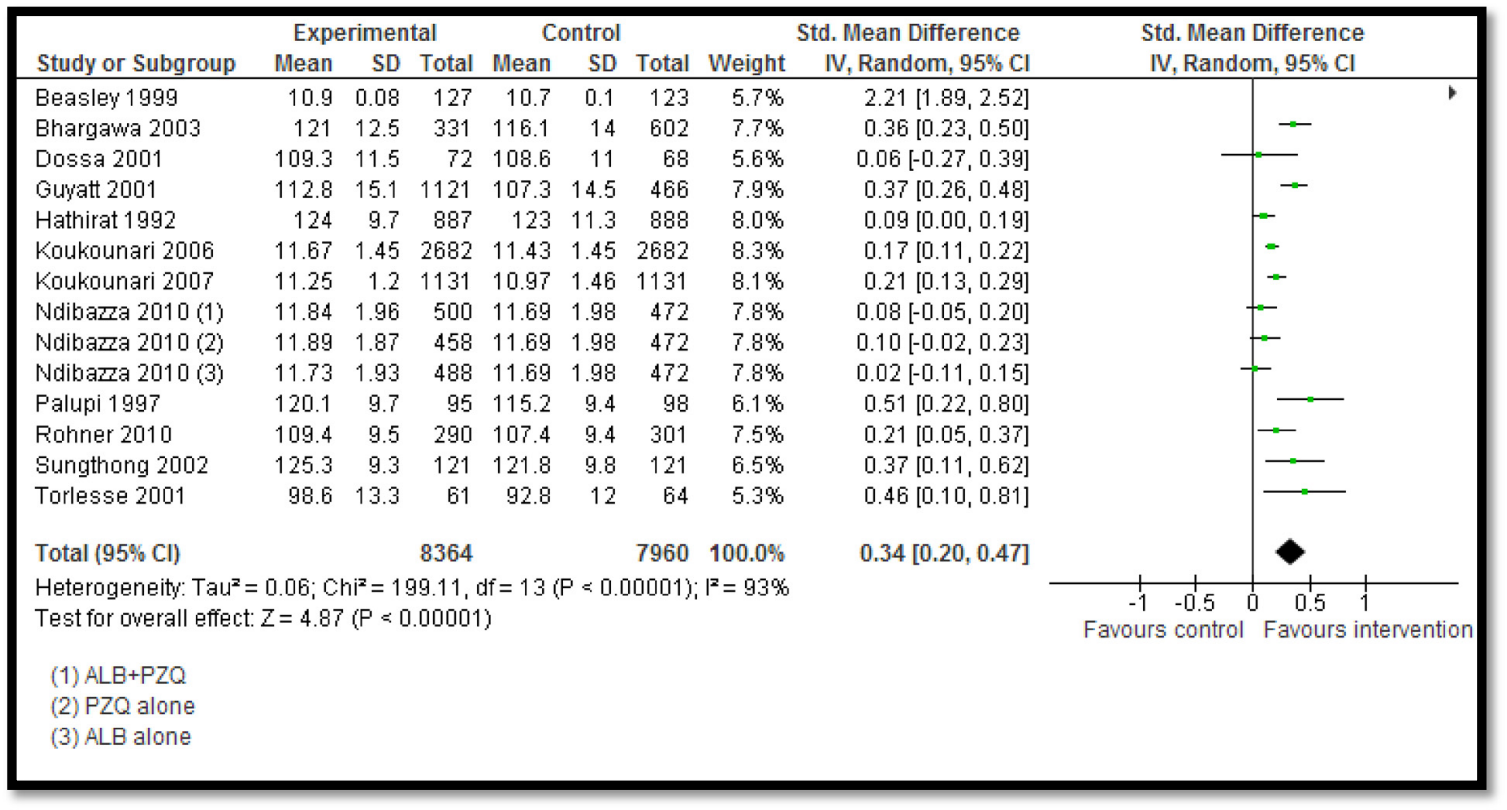
Forest plot for the impact of CBIs on hemoglobin.

**Figure 5:**
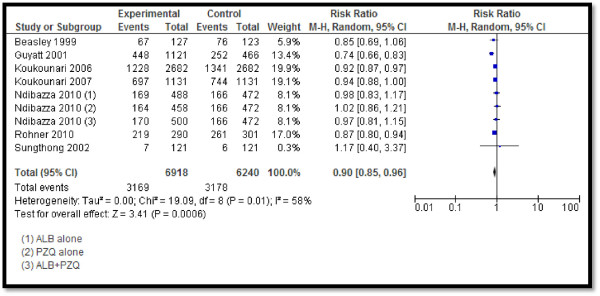
Forest plot for the impact of CBIs on anemia.

Twelve studies reported STH prevalence, showing a significant 53% reduction in the overall STH prevalence (RR: 0.47, 95% CI: 0.41, 0.54) rate, 60% reduction in hookworm prevalence (RR: 0.40, 95% CI: 0.31, 0.52), 68% in ascariasis (RR: 0.32, 95% CI: 0.20, 0.51), and 34% (RR: 0.66, 95% CI: 0.55, 0.80) in trichuriasis prevalence (see Figure 2). Twenty-five studies pooled for schistosomiasis prevalence also showed a significant 59% overall reduction (RR: 0.41, 95% CI: 0.34, 0.50), with 59% (RR: 0.41, 95% CI: 0.30, 0.57), 76% (RR: 0.24, 95% CI: 0.07, 0.80) and 52% (RR: 0.48, 95% CI: 0.36, 0.64) reductions in the prevalence of *Schistosoma haematobium*, *Schistosoma japonicum*, and *Schistosoma mansoni*, respectively (see Figure 3). CBIs also significantly reduced the mean STH intensity (SMD: −3.16, 95 CI: −4.28, −2.04).Twelve studies reported on the hemoglobin (Hb) levels and showed significantly improved mean Hb in the intervention group (SMD: 0.34, 95% CI: 0.20, 0.47), while anemia significantly reduced by 10% (RR: 0.90, 95% CI: 0.85, 0.96) (see Figures 4 and 5). Impacts on serum ferritin, weight, height and delivery outcomes of stillbirth, low birth weight (LBW), and very LBW remained non-significant in the overall, as well as the subgroup, analyses.

Our subgroup analysis for the preventive and therapeutic drug administration showed that preventive drug administration significantly reduced the overall prevalence of STH (RR: 0.43, 95% CI: 0.31, 0.59), STH intensity (SMD: −0.22, 95% CI: −0.26, −0.17), and schistosomiasis prevalence (RR: 0.39, 95% CI: 0.27, 0.55), with non-significant impacts on ascariasis, trichuriasis, and *Schistosoma haematobium* prevalence. It also significantly improved mean Hb (SMD: 0.19, 95% CI: 0.12, 0.26) and reduced anemia prevalence (RR: 0.90, 95% CI: 0.85, 0.96) and VLBW (RR: 0.38, 95% CI: 0.16, 0.87). Preventive chemotherapy did not have a significant impact on serum ferritin, height, weight, LBW, or stillbirths. Therapeutic drug administration showed significant reductions in STH prevalence (RR: 0.52, 95% CI: 0.44, 0.61), intensity (SMD: −5.29, 95% CI: −9.22, −1.36), and schistosomiasis prevalence (RR: 0.41, 95% CI: 0.30, 0.55). Our analysis did not show a significant impact of therapeutic chemotherapy on serum ferritin, Hb, weight, or height.

Findings from the subgroup analysis for school-based, non-integrated, and integrated delivery strategies suggest that school-based delivery significantly reduced STH prevalence (RR: 0.49, 95% CI: 0.39, 0.63) and intensity (SMD: −0.22, 95% CI: −0.26, −0.17), and the prevalence of all types of schistosomiasis (RR: 0.50, 95% CI: 0.33, 0.75), with a non-significant impact on *Schistosoma mansoni*. It also improved mean Hb (SMD: 0.24, 95% CI: 0.16, 0.32) and reduced anemia prevalence (RR: 0.87, 95% CI: 0.81-0.94) among school children. However, school-based delivery did not show any significant impact on serum ferritin, height, and weight. Non-integrated delivery reduced STH prevalence (RR: 0.52, 95% CI: 0.41, 0.67), intensity (SMD: −5.29, 95% CI: −9.22, −1.36), and schistosomiasis prevalence (RR: 0.42, 95% CI: 0.31, 0.57), with a non-significant impact on *Schistosoma mansoni*. Integrated delivery improved schistosomiasis prevalence (RR: 0.24, 95% CI: 0.11, 0.5) and overall STH prevalence (RR: 0.30, 95% CI: 0.12, 0.78), with non-significant impacts on the prevalence of hookworm or trichuriasis.

### Qualitative synthesis

Since most of the quantitative outcomes reported pertained to disease specific indicators, we also attempted to qualitatively synthesize the findings reported in the included studies on other pragmatic parameters from our conceptual framework. CBIs have been reported to achieve large-scale nationwide coverage as seen in Burkina Faso, which was the first country in the WHO African Region to achieve nationwide coverage with anthelminthic drugs against three major NTDs: LF, schistosomiasis, and STH [18]. Furthermore, when delivered in integration with the existing health systems, these programs can achieve maximum coverage as seen in the Gizan region, where a vertically-oriented program, involving mobile teams for schistosome control, failed to achieve the target of 80% population coverage and was costly to sustain. The same program, when later on integrated with the existing PHC, led to a significant increase in the numbers of patients examined and chemotherapy coverage. Similarly in Zimbabwe and Burundi, schistosomiasis and other control programs for NTDs have increasingly been integrated into horizontal PHC systems [23]. The available health infrastructure allows the control strategy to be very well integrated into basic health services, enabling it to be sustained and making it affordable for the national health budget [11]. In a health system that generally enjoys a strong structure from the provincial level down to the community level, successful integration of these services are relatively easier and just require making small modifications to the existing system. However, this process may require more attention in countries where the health service structure is weak. The PHC approach has led to better coverage as compared to the vertical programs, and has been effective in reducing the overall prevalence of helminthic infections.

CBIs have achieved a higher coverage without any increase in implementation costs at the district and health facility levels. At the community level, there is an increase in ‘opportunity costs’ from community implementers who volunteer their time, thus forgoing other remunerative activities. Some of the studies reported no major direct additional costs incurred because the drugs, training materials, and stationary were provided by the PHC from their available resources, and the community health workers (CHWs) were not given any monetary incentives. Hence, the major additional costs included the indirect cost of labour hours for the CHWs and the PHC staff during training and implementation of the program. However, for national scale-up, the biggest challenge is likely to be the cost of training and incentives for the CHWs [27]. Similarly, school-based delivery of preventive and promotive interventions is also reported to be cost effective, however, its success depended on close supervision of the teachers’ compliance with the program implementation, collaboration of the educational authorities, and enthusiastic participation of the school personnel [24]. On the contrary, in some endemic areas, an integrated delivery strategy has not been successful in improving coverage because of the poor access to health services in remote areas. In view of the strong commitment of national health authorities and funding agencies to increase PHC coverage, rapid improvement of this situation can be expected. For sustained reduction in infection prevalence and complete eradication, periodic implementation is also necessary to ensure continuity of morbidity control and achieve universal coverage. In endemic areas, an intermittent intervention delivery has shown to reduce the prevalence but did not completely eradicate the infections. This might be attributable to the fact that chemotherapy reduces disease prevalence only at the time of its administration and does not prevent reinfection [32]. Hence, in endemic areas, periodic implementation along with efforts to prevent reinfection is required.

Most of the studies support a multi-component strategy involving chemotherapy, health education, improved water supply and sanitation, and snail control. Health education and community involvement have been highlighted as essential components in any strategy for helminthic infection control and have been used in many countries [12,22,33]. Examples of such successful programs are the chemotherapy program in Sudan where village health committees served as facilitators, and in Zimbabwe where CHWs were involved in the implementation of improvements to the water supply, sanitation, and in health education programs. Similarly in Ethiopia and Egypt, local health personnel, farmers, school health visitors, and teachers assisted in the implementation of successful control programs. Knowledge and education plays an important role as, apparently, the information motivates the participants and consequently ensures a higher level of compliance [30,40]. The process of designing the program, from its very initiation, should include open discussions and involvement of all partners from the international to the local level. This will ensure that everyone understands the goals, benefits and underlying principles of the project, and have the flexibility needed to adapt to particular local conditions in order to achieve these goals [40]. Strong commitment of all partners to the intervention has reportedly led to excellent participation levels and a significant clinical impact [34,40]. Besides health education and community participation, the socioeconomic development leading to a general rise in living standards, improved sanitation and water supplies, construction of drilled wells in rural areas, and a significant increase in medical care also played a large part in the control of helminthic NTDs [33]. Another important enabling factor was free distribution of drugs and supplies to ensure equitable distribution to poor and marginalized groups, which also helped avoid a complicated system of cost recovery and achieve high compliance levels.

Financial, logistical, and organizational limitations were the major reported constraints in the long-term sustainability of such programs. Despite considerable efforts and their high costs, the results of some of the vector control programs have been disappointing, largely because of poor weed control, lack of maintenance, and the operational difficulty of covering entire networks, which leads to rapid recolonization of treated sites. Large-scale health education programs will not serve their purpose if alternatives to traditional water contact activities are not available. Maintenance is still reported as a major hindrance and local authorities should try to find adequate solutions. Annual tax payments, private latrine programs, subsidized prices, and credit facilities are some of the counter strategies devised to overcome these issues and ensure sustainability of such programs after withdrawal of external funds [11]. Another major challenge is to maintain a high level of participation and enthusiasm as the project is integrated into the routine provincial health program [40]. Although maximum coverage has been achieved in the school-based programs, frequent migration of people (particularly from the fishing communities), influx from neighboring countries, and absence from school of some school-aged children remain to be significant hurdles to the successful implementation of the program [23]. Although often disregarded, religious, economic, and educational heterogeneity within communities may also impair disease control efforts and, hence, these subtle demographic variables must be taken into account.

## Discussion

The evidence in this review comes from efficacy and effectiveness studies on CBIs for helminthic NTDs compared to routine facility-based care or no intervention. Our review findings suggest that CBIs are effective in reducing the prevalence of STH and schistosomiasis, and STH intensity. They are also effective in improving mean Hb and reducing anemia prevalence, but there was non-significant impact on serum ferritin, height and weight gain, LBW, and stillbirths. School-based delivery was identified as a potential medium of delivery as it significantly reduced STH and schistosomiasis prevalence, STH intensity, and anemia prevalence, however, there was limited data available on the effectiveness of the integrated and non-integrated delivery strategies. The qualitative synthesis from the included studies supports community-based delivery strategies and suggests that integrated infection prevention and control measures are more effective in achieving greater coverage compared to the routine vertical delivery, albeit it requires an existing strong healthcare infrastructure. Systematic reviews have been conducted on helminthic NTDs previously, however, their scopes were either limited to a particular infection, drug efficacy or intervention, or the review did not evaluate the effectiveness of community delivery strategies [41-45].

The WHO recommends periodic administration of anthelminthic medicines, mainly ALB and MBZ for STH, PZQ for schistosomiasis, and ivermectin or diethylcarbamazine citrate (DEC) for LF once or twice a year depending on the baseline prevalence of the infection to control morbidity among the population at risk [46].

Health education is an essential component in the prevention and control of helminthiasis. Almost all the studies in this review had a health education component focusing on general hygiene and sanitation along with the recommended drug administration. We did not find any quantifiable data from studies on dracunculiasis, LF, and onchocerciasis to be included in the pooled analysis. Among these diseases, much progress has been made for dracunculiasis and a significant reduction in dracunculiasis prevalence of more than 99% has been achieved since 1989 [47]. Dracunculiasis is successfully at the verge of eradication due to a combination of interventions including community-based surveillance systems, intensified case-containment measures, and access to safe drinking water. Globally, 1.39 billion people still require preventive chemotherapy for LF while 123 million people are at risk of becoming infected with onchocerciasis [46,48]. Programs targeting LF and onchocerciasis are in place in endemic countries, for example, the Global Programme to Eliminate LF and the African Programme for Onchocerciasis. These programs enable resource-limited countries to make medicines freely available and allow easy access to healthcare with a consequent reduction in infection prevalence [49].

Community delivery platforms are increasingly being advocated for the prevention and control of major health issues such as nutrition and childhood infection, and hence could be feasible for helminthic NTDs [50,51]. Under these community platforms, integration of various disease specific programs is being encouraged for onchocerciasis, LF, schistosomiasis, STH, and trachoma [52,53]. The integrated delivery of interventions to control these diseases is more feasible and cost effective as these diseases are endemic in specific geographical pockets where population are mostly co-infected and control mainly involves regular MDA of effective preventive chemotherapy. Therefore, a package of drugs for more than one NTD can be feasibly delivered. Critical factors for successful integration include active support of relevant political and health authorities, a clear understanding by all parties of the health issues, and a simple distribution process. However, there is limited data available to gauge the effectiveness of integrated delivery of these programs along with other health programs including routine ANC and PHC setups [54,55]. School-based delivery has proven to be effective for the management and control of helminthic diseases as it requires a minimum amount of training for school teachers to aid implementation and hence doesn’t require an added workforce, whilst providing vast coverage at low costs [56].

## Conclusion

Current evidence emphasizes that effective community-based strategies exist and deliver a range of preventive, promotive, and therapeutic interventions to combat helminthic NTDs. However, there is a need to conduct high-quality studies on the process of developing and implementing an efficient integrated program as previous global programs have focused on the control of a single NTD through a comprehensive approach. These interventions exist within the current health systems in most of the low- and middle-income countries, but there is a need to implement them on a larger scale especially to reach the unreachable.

## Abbreviations

ANC: Antenatal care; CBI: Community based intervention; CHW: Community health worker; IDoP: Infectious diseases of poverty; LF: Lymphatic filariasis; MDA: Mass drug administration; NTD: Neglected tropical disease; PHC: Primary healthcare; STH: Soil-transmitted helminthiasis; WHO: World health organization

## Competing interests

The authors declare that they have no financial or non-financial competing interests.

## Authors’ contributions

ZAB was responsible for designing and coordinating the review. ZSL and HM were responsible for the data collection, screening of the search results, screening of the retrieved papers against the inclusion criteria, appraising the quality of papers, and abstracting the data. RAS, JKD, and ZSL were responsible for data interpretation and writing the review. ZAB critically reviewed and modified the manuscript. All authors read and approved the final manuscript.

## Authors’ information

Zulfiqar A Bhutta: Founding Director, Center of Excellence in Women & Child Health, The Aga Khan University, Karachi, Pakistan and Robert Harding Chair in Global Child Health & Policy, Center for Global Child Health, Hospital for Sick Children, Toronto, Canada

## Supplementary Material

Additional file 1 Multilingual abstracts in the six official working languages of the United Nations. Click here for file
