# Supplementary material for: Community-based interventions for the prevention and control of helmintic neglected tropical diseases
Source: Infect Dis Poverty. 2014 Jul 31;3:23. doi: 10.1186/2049-9957-3-23 (PMC4128617; doi:10.1186/2049-9957-3-23)
Supplement: Additional file 1 — Multilingual abstracts in the six official working languages of the United Nations. [file 2049-9957-3-23-S1.pdf]

Translation of the abstract into the six official working languages of the United Nations

التدخل المجتمعي من أجل الوقاية من أمراض الداء الديداني المدارية المهملة ومكافحتها

ريحانة أ سلام وحسينة مريديا وجي ك داس و زهرة س لاسي وذو الفقار أ بوتة

ملخص:

نهدف من خلال هذه الوثيقة إلى تحليل منهجي لمدى فعالية التدخلات المجتمعية (CBIs) في الوقاية من الداء الديداني بما في ذلك الداء الديداني المنتقل عن طريق التربة(STH) (داء الصفر والديدان الخطافية وداء المسلكات) و داء الخيطيات اللمفاوية وداء كلابية الذنب و داء التنينات والبلهارسيا. لقد راجعنا بصفة منهجية المؤلفات الصادرة قبل شهر ماي من سنة 2013 بما في ذلك 31 دراسة في هذه المراجعة. وتشير النتائج المستخلصة من التحليل التلوي أن التدخل المجتمعي(CBIs) ناجع في الحد من انتشار الداء الديداني المنتقل عن طريق التربة(STH) (RR: 0.45, 95% CI: 0.38, 0.5) و من انتشار البلهارسيا (schistosomiasis) (RR: 0.40, 95% CI: 0.33, 0.50) ومن حدة الداء الديداني المنتقل عن طريق التربة(STH)(SMD: -3.16, 95 CI: -4.28, -2.04). كما أنه فعال في تحسين معدل الهيموغلوبين(SMD: 0.34, 95% CI: 0.20, 0.47) وفي الحد من انتشار فقر الدم(RR: 0.90, 95% CI: 0.85, 0.96)). لكن ليس لهذه التدخلات أي أثر على الفيريتين والطول و الوزن و انخفاض الوزن عند الولادة (LBW) أو موت الجنين داخل الرحم . لقد ساهمت البرامج داخل المدارس من التخفيض وبشكل ملحوظ من الداء الديداني المنتقل عن طريق التربة(STH) (RR:0.49, 95% CI: 0.39, 0.63) ومن انتشار البلهارسيا (RR: 0.50, 95% CI: 0.33, 0.75) ومن حدة الداء الديداني المنتقل عن طريق التربة(STH) (SMD: -0.22, 95% CI: -0.26, -0.17) ومن انتشار فقر الدم :(RR0.87, 95% CI: 0.81, 0.94). كما يحسن في معدل الهيموغلوبين(SMD: 0.24, 95% CI: 0.16, 0.3). إننا لم نجد دليلا قاطعا وباعتماد الاستنتاج الكمي على النجاعة النسبية لاستراتيجية التدخل سواء كانت متكاملة أو غير متكاملة وذلك بسبب محدودية البيانات المتوفرة حول كل مجموعة فرعية. ومع ذلك ومن خلال نفس الدراسات نجد أن الاستنتاج النوعي يدعم إستراتيجيات التدخل المجتمعي ويقترح أن التدابير المتكاملة للوقاية من العدوى ومكافحتها تكون أنجع في تحقيق تغطية أكبر بالمقارنة مع التدخل العمودي الروتيني وإن كان ذلك يتطلب بنية تحتية قائمة تكون قوية. وتشير الأدلة الحالية على وجود استراتيجيات تدخل ناجعة وتقدم مجموعة من التدخلات الوقائية والتعزيزية

والعلاجية لمحاربة أمراض الداء الديداني المدارية المهملة(NTDs) . لكن هناك حاجة بضرورة تنفيذ وتقييم نجاعة البرامج المتكاملة مع برامج مكافحة المرض القائمة على نطاق واسع وفي جميع أنحاء المناطق خاصة ذات الموارد المحدودة من أجل الوصول لمن يصعب الوصول إليه.

Translated from English version into Arabic by malika2012, through

TRANSLATORS
WITHOUT BORDERS

蠕虫病防控的社区干预

Rehana A Salam, Hasina Maredia, Jai K Das, Zohra S Lassi, Zulfiqar A Bhutta

摘要

本文系统的分析了社区干预对于下列　蠕虫病的防控效果，包括土源性蠕虫病（蛔虫病、钩虫病和鞭虫病）、淋巴丝虫病、盘尾丝虫病、麦地那龙线虫病和血吸虫病。我们系统地回顾了 2013 年 5 月以前发表的文献并最终纳入 31 项研究进行分析。Meta 分析显示社区干预能够降低土源性蠕虫病患病率(RR: 0.45, 95% CI: 0.38, 0.54)、血吸虫病患病率 (RR: 0.40, 95% CI: 0.33, 0.50) 和土源性蠕虫病感染度(SMD: -3.16, 95 CI: -4.28, -2.04)。社区干预也能够改善血红蛋白水平(SMD: 0.34, 95% CI: 0.20, 0.47)，降低贫血患病率(RR: 0.90, 95% CI: 0.85, 0.96)，但是对铁蛋白、身高、体重、低出生体重和死产没有影响。以学校为基础的干预能够显著降低土源性蠕虫病患病率(RR: 0.49, 95% CI: 0.39, 0.63)、血吸虫病患病率 (RR: 0.50, 95% CI: 0.33, 0.75)、土源性蠕虫病感染度(SMD: -0.22, 95% CI: -0.26, -0.17)和贫血患病率(RR: 0.87, 95% CI: 0.81, 0.94)。以学校为基础的干预同样能够改善血红蛋白水平(SMD: 0.24, 95% CI: 0.16, 0.32)。由于各个亚组数据有限，在关于综合的和非综合的防控措施效果差异方面未得出任何的定量结论。但是，定性分析表明社区干预策略和综合的防控措施更加有效，因为这样能够达到更高的覆盖率，当然这需要基于强有力的医疗基础设施。现有的证据表明已存在有效的社区干预策略，以一系列预防性的、促进性的化疗手段来抗击以上蠕虫病。但是仍然需要在资源有限地区、特别是在那些以前无法达到的地区，在更大的范围内，对已有的疾病控制项目进行综合规划并加以评价。

Translated from English version into Chinese by Qian Men-bao, through

CHINESE CENTER FOR DISEASE CONTROL AND PREVENTION
NATIONAL INSTITUTE FOR PARASITIC DISEASES

Les interventions communautaires dans la prévention et la lutte contre les helminthiases tropicales négligées

Rehana A Salam, Hasina Maredia, Jai K Das, Zohra S Lassi, Zulfiqar A Bhutta

Résumé

Les auteurs cherchent à analyser, suivant une approche systématique, l'efficacité des interventions communautaires (IC) pour la prévention et la lutte contre les helminthiases, notamment les géohelminthiases (helminthiases transmises par le sol ou HTS : ascaridiase, ankylostomiase et trichocéphalose), la filariose lymphatique, l'onchocercose, la dracunculose et la schistosomiase. Nous avons procédé à une revue systématique de la littérature publiée avant mai 2013 et avons inclus 31 études dans cette revue. Les résultats de cette méta-analyse suggèrent que les interventions communautaires sont efficaces pour réduire la prévalence des géohelminthiases (RR : 0,45, IC 95 % : 0,38, 0,54) et de la schistosomiase (RR : 0,40, IC 95 % : 0,33, 0,50), ainsi que l'intensité des HTS (ET : -3,16, IC 95 % : -4,28, -2,04). Elles sont également efficaces pour améliorer le taux d'hémoglobine moyen (ET : 0,34, IC 95 % : 0,20, 0,47) et réduire la prévalence de l'anémie (RR : 0,90, IC 95 % : 0,85, 0,96). Ce type d'intervention n'a cependant pas eu d'effet sur le taux de ferritine, la taille, le poids, l'hypotrophie néonatale ou la mortinatalité. Les actions en milieu scolaire ont permis de réduire significativement la prévalence des géohelminthiases (RR : 0,49, IC 95 % : 0,39, 0,63) et de la schistosomiase (RR : 0,50, IC 95 % : 0,33, 0,75), l'intensité des HTS (ET : -0,22, IC 95 % : -0,26, -0,17) et la prévalence de l'anémie (RR : 0,87, IC 95 % : 0,81, 0,94). Elles ont également amélioré le taux moyen d'hémoglobine (ET : 0,24, IC 95 % : 0,16, 0,32). La synthèse quantitative des études incluses n'a pas apporté de preuves concluantes sur l'efficacité relative des stratégies intégrées et non intégrées, en raison de la quantité de données limitée disponible pour chaque sous-groupe. Toutefois, elle est favorable aux stratégies de prévention communautaires et suggère que les mesures intégrées de prévention et de lutte contre les infections permettent plus efficacement une couverture étendue que les actions verticales de routine, quoiqu'elles nécessitent une solide infrastructure sanitaire existante. Les preuves actuellement disponibles suggèrent qu'il existe des stratégies communautaires efficaces, qui permettent de réaliser diverses interventions de prévention, de promotion et de traitement pour lutter contre les helminthiases tropicales négligées. Il reste toutefois, pour atteindre l'inatteignable, à appliquer et à évaluer à plus grande échelle des

programmes efficaces, intégrés dans les programmes de prévention des maladies existants dans les régions où les ressources sont limitées.

Translated from English version into French by Suzanne Assenat, through

TRANSLATORS
WITHOUT BORDERS

Влияние вмешательства общины на профилактику и контроль забытых тропических болезней, вызванных гельминтами

Рехана А. Салам, Хасина Маредиа, Джай К. Дас, Зохра С. Ласси, Зульфикар А. Бхутта

Аннотация

В данной работе мы стремимся осуществить систематический анализ эффективности вмешательства общины (ВО) на профилактику и контроль гельминтозов, в том числе геогельминтозов (ГГ: аскаридоза, анкилостомоза и трихиуриаза), лимфатического филяриоза, онхоцеркоза, дракункулеза и шистосомоза. Мы систематически просмотрели литературу, изданную до мая 2013 г., и включили в данный обзор 31 исследование. Результаты мета-анализа показывают, что ВО имеет высокий эффект в снижении распространенности ГГ (отношение рисков: 0,45, 95% ВО: 0,38, 0,54), шистосомоза (отношение рисков: 0,40, 95% ВО: 0,33, 0,50), а также интенсивности ГГ (величина эффекта: -3,16, 95 ВО: -4,28, -2,04). Оно также эффективно для улучшения среднего значения гемоглобина (величина эффекта: 0,34, 95% ВО: 0,20, 0,47) и сокращения распространенности анемии (отношение рисков: 0,90, 95% ВО: 0,85, 0,96). Тем не менее, этот тип вмешательств не имел никакого эффекта на ферритин, рост, вес, низкую массу тела при рождении (НМТР) или количество мертворожденных детей. Программы, проведенные в школах, значительно снизили распространение ГГ (отношение рисков: 0,49, 95% ВО: 0,39, 0,63) и шистосомоза (отношение рисков: 0,50, 95% ВО: 0,33, 0,75), интенсивность ГГ (величина эффекта: -0,22, 95% ВО: -0,26, -0,17), а также распространение анемии (отношение рисков: 0,87, 95% ВО: 0,81, 0,94). Они также улучшили среднее значение гемоглобина (величина эффекта: 0,24, 95% ВО: 0,16, 0,32). Мы не нашли никаких убедительных свидетельств на основе количественного синтеза об относительной эффективности интегрированных и не интегрированных стратегий ведения программ из-за ограниченного объема доступных данных о каждой подгруппе. Тем не менее, качественный синтез включенных в обзор исследований поддерживает стратегии осуществления программ с участием общины и предполагает, что интегрированные меры по профилактике и контролю инфекций более эффективны для охвата большего количества человек по сравнению с обычным вертикальным проведением

программ, хотя это и требует существования сильной инфраструктуры здравоохранения. Доступные в настоящее время данные предполагают, что эффективные стратегии с участием общины существуют и осуществляют ряд профилактических, содействующих и терапевтических вмешательств для борьбы с забытыми тропическими болезнями (ЗТБ), вызванными гельминтами; тем не менее, существует необходимость введения и оценки эффективных интегрированных программ вместе с существующими программами профилактики болезней в более крупном масштабе в регионах с ограниченными ресурсами, особенно для того, чтобы достичь недостижимых областей.

Translated from English version into Russian by Mayya Shlyakhter, through

TRANSLATORS
WITHOUT BORDERS

Intervenciones basadas en la comunidad para la prevención y control de enfermedades tropicales helmínticas descuidadas

Rehana A. Salam, Hasina Maredia, Jai K. Das, Zohra S. Lassi, Zulfiqar A. Bhutta

Extracto

En este trabajo, nuestro objetivo es analizar sistemáticamente la efectividad de las intervenciones de base comunitaria (IBC) para la prevención y el control de la helmintiasis, incluyendo las helmintiasis transmitidas por el suelo (HTS) (ascariasis, anquilostomiasis y tricuriasis), la filariasis linfática, la oncocercosis, la dracunculiasis y la esquistosomiasis. Llevamos a cabo una revisión sistemática de la literatura publicada antes de mayo de 2013 y se incluyeron 31 estudios en esta revisión. Los resultados del meta análisis sugieren que las IBC son eficaces para reducir la prevalencia de las HTS (RR: 0.45, IC 95%: 0,38, 0,54), la esquistosomiasis (RR: 0,40, IC 95%: 0,33, 0,50), y la intensidad HTS (DME: -3,16, IC del 95: -4,28, -2,04). También son eficaces para mejorar la hemoglobina media (DME: 0,34, IC del 95%: 0,20, 0,47) y reducir la prevalencia de anemia (RR: 0,90, IC 95%: 0,85, 0,96). Sin embargo, este tipo de intervenciones no tuvo ningún impacto en la ferritina, altura, peso bajo al nacer (PBN) o nacidos muertos. La entrega con base en la escuela reduce significativamente las HTS (RR: 0,49, IC 95%: 0,39 a 0,63) y la prevalencia de la esquistosomiasis (RR: 0,50, IC 95%: 0.33, 0.75), la intensidad de las HTS (DME: -0,22, IC del 95%: - 0,26, -0,17), y la prevalencia de anemia (RR: 0,87, IC 95%: 0,81, 0,94). También mejoró la hemoglobina media (DME: 0,24, IC del 95%: 0,16, 0,32). No se encontró ninguna prueba concluyente de la síntesis cuantitativa de la eficacia relativa de las estrategias integradas y no integradas en las entregas debido a la escasez de datos disponibles para cada subgrupo. Sin embargo, la síntesis cualitativa de los estudios incluidos apoya las estrategias de las entregas basadas en la comunidad y sugiere que las medidas integradas de prevención y control de las infecciones son más eficaces en el logro de una mayor cobertura en comparación con la entrega vertical de rutina, aunque requiere la existencia de una sólida infraestructura sanitaria. La evidencia actual sugiere que existen estrategias eficaces de base comunitaria y que ofrecen una gama de intervenciones preventivas, de promoción y terapéuticas para combatir las enfermedades helmínticas tropicales desatendidas (ETD); sin embargo, existe la necesidad de aplicar y evaluar programas eficientes e integrados con los programas existentes de control de las enfermedades en

una escala mayor en todas las regiones con recursos limitados, especialmente para llegar a las inalcanzables.

Translated from English version into Spanish by Susana Rosselli, through

TRANSLATORS
WITHOUT BORDERS
